# Simultaneous Functional Magnetic Resonance and Optoacoustic Imaging of Brain‐Wide Sensory Responses in Mice

**DOI:** 10.1002/advs.202205191

**Published:** 2022-11-27

**Authors:** Zhenyue Chen, Irmak Gezginer, Mark‐Aurel Augath, Yu‐Hang Liu, Ruiqing Ni, Xosé Luís Deán‐Ben, Daniel Razansky

**Affiliations:** ^1^ Institute for Biomedical Engineering and Institute of Pharmacology and Toxicology Faculty of Medicine University of Zurich Zurich 8057 Switzerland; ^2^ Institute for Biomedical Engineering Department of Information Technology and Electrical Engineering ETH Zurich Zurich 8093 Switzerland; ^3^ Zurich Neuroscience Center (ZNZ) Zurich Switzerland

**Keywords:** brain activation, electrical paw stimulation, functional magnetic resonance imaging, multimodal imaging, multispectral optoacoustic tomography, neuroimaging

## Abstract

Functional magnetic resonance imaging (fMRI) has massively contributed to the understanding of mammalian brain function. However, the origin and interpretation of the blood oxygen level‐dependent (BOLD) signals retrieved by fMRI remain highly disputed. This article reports on the development of a fully hybridized system enabling concurrent functional magnetic resonance optoacoustic tomography (MROT) measurements of stimulus‐evoked brain‐wide sensory responses in mice. The highly complementary angiographic and soft tissue contrasts of both modalities along with simultaneous multi‐parametric readings of stimulus‐evoked hemodynamic responses are leveraged in order to establish unequivocal links between the various counteracting physiological and metabolic processes in the brain. The results indicate that the BOLD signals are highly correlated, both spatially and temporally, with the total hemoglobin readings resolved with volumetric multi‐spectral optoacoustic tomography. Furthermore, the differential oxygenated and deoxygenated hemoglobin optoacoustic readings exhibit superior sensitivity as compared to the BOLD signals when detecting stimulus‐evoked hemodynamic responses. The fully hybridized MROT approach greatly expands the neuroimaging toolset to comprehensively study neurovascular and neurometabolic coupling mechanisms and related diseases.

## Graphical abstract

A fully hybridized magnetic resonance optoacoustic tomography system is devised for concurrent multi‐modal measurement of brain‐wide sensory responses in mice. Blood oxygen level‐dependent signal, oxyhemoglobin, total hemoglobin, and oxygen saturation are simultaneously measured in response to peripheral stimuli to allow for a comprehensive study of neurovascular and neurometabolic coupling mechanisms in the brain.

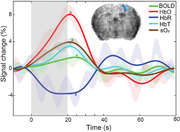

## 1 Introduction

Functional magnetic resonance imaging (fMRI) is an indispensable tool for neuroscience, owing to its whole‐brain imaging capacity, excellent anatomical contrast, and spatial resolution.^[^ ^1^, ^2^ ^]^ The small size of the mouse brain yet makes it challenging to measure brain activity with adequate signal‐to‐noise ratio (SNR) levels whilst maintaining a small voxel size and short scan times. This commonly leads to hard trade‐offs between the effective field‐of‐view, and spatial and temporal resolution of the technique.^[^ ^3^ ^]^ Due to the existing delay between brain activation onset and the peak of the blood oxygen level‐dependent (BOLD) signal, fMRI also has a limited ability to capture fast neural responses occurring in the brain.^[^ ^4^ ^]^ More importantly, the complicated origin of the BOLD signal being affected by multiple factors, such as blood flow, blood volume, metabolic rate of oxygen, and baseline physiological state, hinders unequivocal interpretation of the fMRI readings.^[^ ^5^ ^]^ Furthermore, label‐free fMRI contrast suffers from a lack of sensitivity to molecular and cellular targets in the brain, making it challenging to visualize distinct molecular hallmarks of neural activity. Although various molecular fMRI approaches have been proposed to fill the gap, considerable hurdles remained in the design of suitable probes to enable sufficient sensitivity for fast, high‐resolution data acquisitions.^[^ ^6^ ^]^

**Figure 1:**
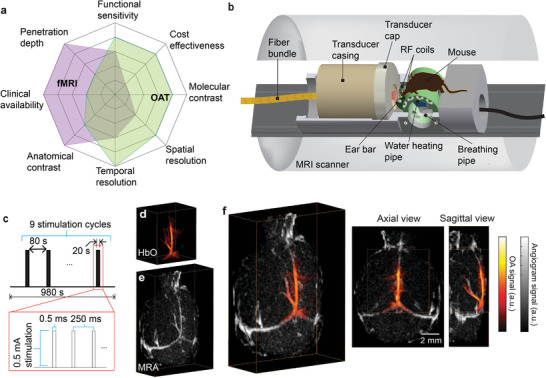
Hybrid system for concurrent magnetic resonance optoacoustic tomography (MROT) imaging of murine brain activation. a) Illustration showing the imaging performance comparison between fMRI and OAT and the high complementary value of their combination for functional neuroimaging studies. b) Schematics of the hybrid MROT system featuring the 9.4T MRI bore inserted with a customized MRI‐compatible spherical matric array transducer for volumetric data acquisition, a fiber bundle for pulsed light delivery, animal fixation parts, and radiofrequency (RF) coils. c) Electrical stimulation paradigm applied to the left forepaw of a mouse. Stimulation cycle parameters: 0.5 ms pulse duration, 0.5 mA current, 4 Hz pulse repetition frequency, 20 s duration. One stimulation sequence consists of nine stimulation cycles repeated every 100 s. d) Volumetric OAT image of the mouse brain (HbO component is shown). e) The corresponding magnetic resonance angiography (MRA) image acquired with the fast low angle shot (FLASH) sequence. f) Axial and sagittal views of the coregistered OAT and MRA images.

Optoacoustic imaging can probe rich and versatile optical contrast across a wide domain of penetration scales into optically dense tissues while maintaining excellent spatio‐temporal resolution representative of ultrasound imaging. The technique is ideally suited for high‐resolution label‐free vascular imaging with a multitude of applications pursued in the areas of cardiovascular and brain research, oncology, and more.^[^ ^7^, ^8^, ^9^, ^10^, ^11^ ^]^ Multi‐spectral optoacoustic tomography (OAT) has been shown capable of visualizing brain‐wide neuronal activity in mice to record stimulus‐evoked hemodynamics, disease‐related brain signaling, as well as fast calcium responses in 3D with high spatial resolution in the 100 µm range and millisecond‐level temporal precision.^[^ ^11^, ^12^, ^13^ ^]^ Latest progress in OAT has enabled real‐time mapping of hemodynamic changes in the human brain with high spatio‐temporal resolution.^[^ ^14^ ^]^ The powerful spectroscopic imaging capacity of OAT is highly suitable for multi‐parametric label‐free characterization of brain hemodynamics via simultaneous mapping of multiple hemodynamic parameters such as oxyhemoglobin (HbO), deoxyhemoglobin (HbR), total hemoglobin (HbT), and blood oxygen saturation (sO_2_).^[^ ^15^, ^16^ ^]^ These readings can significantly augment and supplement the information resolvable by fMRI, which is primarily related to HbR changes.^[^ ^17^ ^]^ On the other hand, optoacoustic imaging provides limited soft tissue contrast mainly stemming from vascular structures, which can be compensated by the excellent anatomical contrast provided by MRI, further underscoring the highly complementary nature of these two modalities for functional neuroimaging (**Figure** **1**a).

Hybridization of OAT and MRI for concurrent measurements in rodents is a challenging task with multiple technological issues to be addressed pertaining to magnetic compatibility, hardware complexity and synchronization, mutual signal interference, physical constraints of the MRI bore, and in vivo compatibility of the multi‐modal environment. The previous study has shown the general feasibility of sequentially acquiring static images from tissue phantoms with an integrated approach,^[^ ^18^ ^]^ which was however not suitable for dynamic concurrent 3D image acquisitions from living animals. Here, we report on the successful implementation of a fully hybridized system enabling simultaneous functional magnetic resonance optoacoustic tomography (MROT) measurements of stimulus‐evoked brain‐wide sensory responses in living mice. The volumetric OAT imaging module was designed as an insert into a high‐field MRI scanner by integrating a customized MRI‐compatible spherical transducer array, illumination bundle, and radiofrequency (RF) coil into a 3D‐printed mouse holder (Figure 1b). A dedicated image processing pipeline was further developed for image reconstruction, spectral unmixing, multi‐modal image registration, and statistical parametric mapping (SPM) to facilitate the analysis of the hybrid MROT functional data.

## 2 Results

The hybrid MROT system was designed such that the light delivery and ultrasound detection array modules can be operated inside a 9.4T preclinical MRI scanner (Figure S1, Supporting Information). The platform inserted in the MRI bore consisted of a customized MRI‐compatible spherical matrix array transducer for volumetric data acquisition, a fiber bundle for pulsed light delivery, animal fixation parts, and RF coils (Figure 1b). Due to the limited space inside the scanner bore, all components in the platform were confined within a cylinder with a diameter of 110 mm. The animal was placed in a prone position on a cylindrical platform (mouse bed) with its nose facing down. A plastic pipe flushed with warm water was inserted through the transversal apertures of the mouse bed to maintain the body temperature during data acquisition. Two loops of a saddle RF coil were integrated on either side of the mouse head holder with polarization perpendicular to the main magnetic field, as required for efficient MRI acquisitions. For recording stimulus‐evoked responses, the simultaneous MROT data acquisition lasting 980 s was synchronized with a unilateral electrical forepaw stimulation paradigm (Figure 1c). This was achieved by externally triggering the initiation of the MRI acquisition protocol and the electric stimulator with the first laser pulse.

While image distortions for the various MRI sequences were successfully mitigated by devising customized MRI‐compatible transducer and illumination bundle, as well as heavy water for acoustic coupling, the raw recorded OAT sinograms were more significantly affected by the concurrent MRI acquisitions resulting in corrupted signals distributed randomly across the transducer channels and different time instances (Figure S2, Supporting Information). This has been addressed by developing a dedicated sinogram restoration algorithm (see Experimental Section for details), which ensured the generation of artifact‐free time‐lapse volumetric OAT data (Figure S3, Supporting Information). Major cerebral vessels could clearly be identified both in the HbO component unmixed from the multi‐spectral OAT data (Figure 1d), as well as the magnetic resonance angiography (MRA) images acquired with the fast low‐angle shot (FLASH) sequence (Figure 1e). Those enabled accurate image coregistration between the two modalities with a dedicated protocol (Figure 1f). In this way, T1‐weighted and BOLD fMRI images could be accurately superimposed onto the simultaneously acquired OAT images for further analysis.

**Figure 2:**
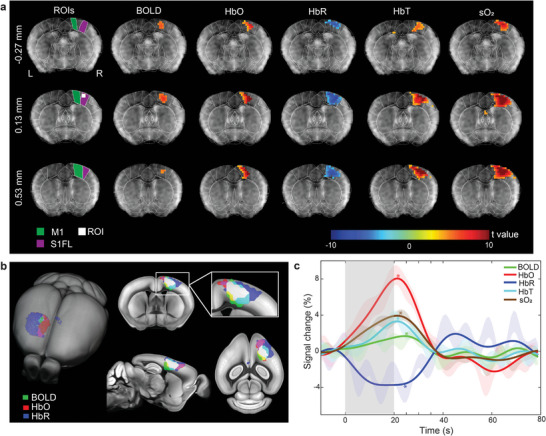
Representative stimulus‐evoked responses recorded by MROT. a) Multi‐slice (volumetric) activations maps of the BOLD, HbO, HbR, HbT, and sO_2_ hemodynamic components overlaid to the anatomical T1‐weighted image of the brain. Statistically significant responses, as resolved by the general linear model of the SPM12 software with one‐sided *t*‐tests, were observed in the S1FL and M1 brain areas. The brain regions in the coronal slices were segmented based on the Allen Mouse Brain Common Coordinate Framework (CCF). The left side of the image corresponds to the left hemisphere. b) Superposition of the BOLD, HbO, and HbR activation maps onto the mouse brain atlas. The white color indicates the overlapping region of the three components. c) Averaged fractional signal intensity changes of the hemodynamic components following the sensory stimulation. The activation time curve for each component was computed from a 0.4 × 0.4 × 0.4 mm^3^ region of interest located in the contralateral S1FL brain area, as labeled in panel (a). Time‐to‐peak (TTP) values are indicated with * for different components. Shaded regions show standard error of mean (SEM) across all the stimulation sequences in different animals. The grey bar indicates the stimulation period.

The functional multi‐modal data were pre‐processed and further analyzed with the general linear model (GLM) using the SPM12 software. This enabled calculating the activation maps for all the resolved hemodynamic components, namely, BOLD for fMRI and HbO, HbR, HbT, and sO_2_ for OAT. Statistically significant activation (family‐wise error [FWE] corrected, *p* < 0.05) in the contralateral primary somatosensory cortex forelimb area (S1FL) and primary motor cortex (M1) regions was observed across all the components (**Figure** **2**a), while no statistically significant activation was detected in the corresponding ipsilateral brain regions (Figure S4, Supporting Information). Similar patterns in the activation maps were observed in all animals (*n* = 6) measured in this study (Figure S5, Supporting Information). Superposition of the activation maps of BOLD, HbO, and HbR revealed a high spatial correlation between the stimulus‐evoked activated areas observed in cross sections of the volumetric multi‐modal functional data (Figure 2b). The activation time courses for each hemodynamic component from a 0.4 × 0.4 × 0.4 mm^3^ region of interest (ROI) within the contralateral S1FL region (Figure 2a) resembled oscillations at characteristic 0.01 Hz frequency that matched the boxcar frequency of the stimulation paradigm (Figures S6 and S7, Supporting Information). The extracted signals were subsequently averaged for all the stimulation cycles to compute the time traces and fractional signal changes of each component. The BOLD, HbO, HbT, and sO_2_ signal intensities increased by 1.62%, 7.99%, 3.25%, and 3.88%, respectively, while the HbR signal intensity decreased by 3.79% following the sensory stimulation (Figure 2c) in congruence with previous reports that employed stand‐alone functional neuroimaging modalities.^[^ ^19^, ^20^ ^]^ The strongest fractional signal change was observed in the HbO component. This manifests the added value of OAT imaging as changes in HbO cannot be readily discerned from the BOLD signals. On the other hand, variable time‐to‐peak (TTP) values were observed across different components with their peaks falling into a time window within 2–5 s after the stimulation onset, which is consistent with previous findings.^[^ ^21^ ^]^ Note that the BOLD signal is known to primarily reflect changes in HbR whereas the positive BOLD response corresponds to a declining HbR level, which agrees with the independently acquired OAT measurements. The delay in HbR relative to initial changes in HbO and HbT is arguably attributed to the wash‐in oxygenated blood during hyperemia. More significantly, the undershoot of the BOLD response corroborates the overshoot of HbR post‐stimulation and is correlated to a reduction of the cerebral blood volume with the oxygen saturation remaining unaltered. This evinces that the BOLD post‐stimulus response is in fact a hemodynamic rather than a metabolic phenomenon.^[^ ^22^, ^23^ ^]^ Interestingly, periodic fluctuations with ≈0.1 Hz frequency were observed most prominently in the HbR channel (Figure S7, Supporting Information), which is ascribed to increased rhythmicity of cortical activity under ketamine‐xylazine anesthesia.^[^ ^24^ ^]^

**Figure 3:**
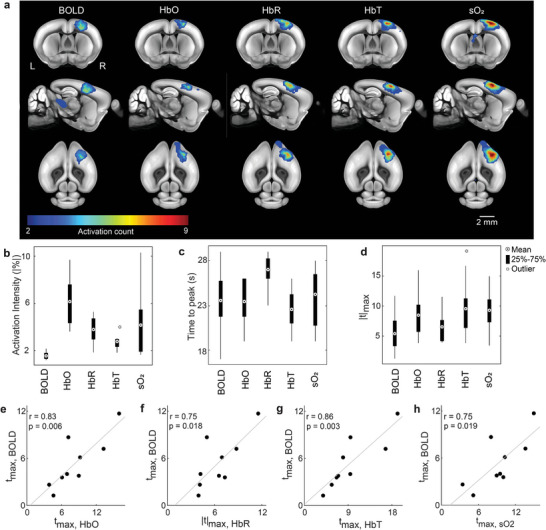
Group‐level analysis of responses to the sensory stimulation. a) Activation count maps for the different hemodynamic components. Values indicate the frequency of statistically significant activation events for each voxel (nine sequences, *n* = 6 mice). Count maps are overlaid onto the mouse brain atlas. b,c) Group‐level statistics on the averaged time‐courses from the S1FL brain region. The distribution of activation intensity and TTP values calculated from the time courses of different hemodynamic components are displayed. d) Group‐level statistics on the maximum *t*‐value (*t*_max_) from the S1FL region. The distribution of *t*_max_ values is displayed for different components. e–h) Scatter plots showing the relationship between *t*_max_ values. Dots represent individual stimulation sequences. Pearson correlation coefficients (*r*) were calculated for the plots. Grey lines indicate linear fits for the scatter plots.

The spatial and temporal correlation among the responses measured for different hemodynamic components was subsequently investigated for nine sequences (each sequence lasted 980 s including nine individual stimulation cycles) recorded from different mice (*n* = 6) that were stimulated and imaged with the same protocol (Figure 1c). Activation count maps, defined by the number of detected significant activation responses from each image voxel across all nine sequences, were then computed for each hemodynamic component. Although all the components exhibited well‐localized activation in contralateral S1FL and M1 regions, the largest number of significant activation counts appeared in the HbT and sO_2_ readings indicating a generally higher sensitivity of these components to the stimulation paradigm (**Figure** **3**a). Statistics on activation intensity, as well as TTP and maximum *t*‐values, were further performed for all the components in the S1FL brain region across all the stimulation trials (Figure 3b–d). Stronger activation was observed in all the hemodynamic components recorded by OAT versus the BOLD readings (1.64 ± 0.12% (SEM)). The highest fractional change was observed in the HbO component (6.22 ± 0.76%), followed by sO_2_ (4.22 ± 1.04%), HbR (−3.87 ± 0.41%), and HbT (2.80 ± 0.23%) (Figure 3b). Variable TTP values with comparable mean values were manifested in the HbO (23.44 ± 0.85 s), sO_2_ (24.22 ± 1.14 s), HbT (22.56 ± 0.73 s), and BOLD (23.56 ± 1.20 s) components, while a longer TTP was observed in HbR (26.89 ± 0.63 s) with respect to the other components (Figure 3c). Likewise, the OAT readings exhibited higher maximum *t*‐values as compared to BOLD (5.44 ± 1.10) in the contralateral S1FL region. The highest maximum *t*‐values were observed for HbT (9.52 ± 1.71), followed by sO_2_ (9.33 ± 1.21), HbO (8.45 ± 1.32), and HbR (6.49 ± 0.84) (Figure 3d). Further comparison of *t*_max_ values revealed the highest correlation between BOLD‐HbT (*r* = 0.86, Figure 3g) followed by BOLD‐HbO (*r* = 0.83, Figure 3e), BOLD‐HbR (*r* = 0.75, Figure 3f), and BOLD‐sO_2_ (*r* = 0.75, Figure 3h) while the comparison of activation intensity and TTP revealed a relatively loose correlation (Figures S8 and S9, Supporting Information).

## 3 Discussion

In this work, we report on the development and in vivo validation of a fully hybrid MROT system for concurrent multi‐modal measurement of brain‐wide sensory responses in mice. Synchronized upsurge in the BOLD, HbO, HbT, and sO_2_ hemodynamic components along with an HbR signal decrease were observed upon sensory stimulation. Noticeably, HbT and sO_2_ components retrieved by the OAT modality exhibited superior sensitivity in detecting stimulus‐evoked hemodynamic responses. Among the different hemodynamic readings, the BOLD signals have been found to be tightly correlated to HbT, thus corroborating previous findings on the hemodynamic mechanisms underlying the fMRI signal.^[^ ^25^ ^]^ Although electrical paw stimulation paradigms have been broadly applied in rodent brain studies, the reported somatosensory responses in anesthetized mice are often not well localized and inconsistent among the different studies, with most reports evincing ipsilateral activation and pain‐like widespread activation patterns.^[^ ^26^, ^27^ ^]^ In this work, we adopted a relatively mild electrical paw stimulation paradigm. Thus, except for toe twitching upon stimulation, no body tremble nor other abnormal motion was observed from the animal. Our work revealed highly correlated well‐confined responses in the mouse S1FL cortex region. Out of nine stimulation sequences performed in different mice, one sequence resulted in no significant activation in the BOLD nor the corresponding HbR component, while another sequence had led to no significant activation in BOLD yet significant activation in all the other hemodynamic components recorded by OAT. The relatively low sensitivity and high variability of the BOLD responses evinced again the complex origin of the BOLD signal. In contrast, analysis of the HbO, HbT, and sO_2_ components consistently resulted in statistically significant activation in response to sensory stimulation in all the stimulation sequences. Interestingly, HbO, HbT, and sO_2_ components displayed weak activation in the sequences where the BOLD or HbR components exhibited no significant activation. While heterogeneous brain responses across different animals are to be expected, the BOLD and HbR hemodynamic components are generally less sensitive in detecting stimulus‐evoked hemodynamic responses. Nevertheless, localized contralateral activation without widespread pain‐like response was manifested across all the components. Note that the impact on the hemodynamic response of the unconventional position of the animal was assessed by comparing standalone OAT measurements where the animal lay flat and no obvious difference in the response was observed.

Note that anesthesia can alter neural activity and brain function as compared to experiments performed in awake animals. Unlike rats, fMRI activity in mice under a‐chloralose or isoflurane anesthesia appeared to be unspecific.^[^ ^27^, ^28^ ^]^ In this work, we adopted instead a previously reported intraperitoneal (IP) ketamine/xylazine (K/X) cocktail anesthesia protocol for fMRI in murine models.^[^ ^26^ ^]^ The K/X cocktail exhibited a robust, longitudinal cortical response to forepaw stimulation in high‐field (9.4T) MRI, which was confirmed in our study. The most recent study on the characterization of somatosensory activity in mice under dexmedetomidine/isoflurane and K/X anesthesia revealed that both anesthetics could provide a brain‐wide, robust, and stable BOLD response throughout the somatosensory axis for up to 8 h.^[^ ^29^ ^]^ In our work, data acquisition was performed within the first 2 h after induction of the anesthesia.

Compared with other possible multimodal combinations, hybridization between OAT and MRI is highly advantageous for a number of reasons: 1) Sound validation of the relatively new OAT‐based neuroimaging tools remains challenging, which could potentially be addressed by concurrent imaging with the well‐established fMRI methods; 2) Both modalities can provide volumetric dynamic information on comparable spatial scales with MRI further providing excellent soft tissue contrast that can compensate for the lack of brain anatomical landmarks in OAT images; 3) The superior spatio‐temporal resolution performance of OAT is complemented by the deeper penetration depth of fMRI; 4) The direct measurement of different hemodynamic components by OAT can facilitate better interpretation of the BOLD signal, which is intrinsically linked to several, often counteracting, physiological and metabolic processes in the brain. Despite the widespread use of fMRI in the last two decades, the origin of the BOLD signal is still poorly understood. The classical interpretation of a positive BOLD response assumes that an untick in neuronal activity generates a proportional increase in the local blood volume and flow resulting in increased local oxygenation levels with decreasing HbR.^[^ ^30^ ^]^ However, the relationship between BOLD and brain metabolism is less obvious, which is manifested by the pronounced and long‐lasting undershoot in the post‐stimulus BOLD signals. Owing to the multiparametric assessment of hemodynamic responses enabled by MROT, both the blood flow responses and the metabolic rate of oxygen consumption can be recorded simultaneously. Our results indicate that the combination of reduced HbT and unaltered sO_2_ evinces that the BOLD undershoot post‐stimulation is attributed to a hemodynamic rather than a metabolic response of the brain. However, more delicate experimental designs are required to fully decipher the BOLD responses and their underlying physiological and metabolic processes.

Mutual interference between the OAT and MRI is inevitable as the 9.4 T magnetic field and rapidly changing gradients form a hostile working environment for any sophisticated electronics.^[^ ^18^ ^]^ On the other hand, the inserted electronic components along with water and ultrasound gel required for optimal acoustic coupling in OAT recordings may distort the magnetic fields used for MRI. By devising a customized MR‐compatible transducer and illumination bundle, as well as heavy water for coupling, negligible image distortions were observed for various MRI sequences. In contrast, the raw recorded OAT sinograms were more significantly affected by the concurrent MRI acquisitions, which has been addressed via a dedicated signal restoration algorithm. A similar approach can be adopted for combining other imaging modalities with MRI, such as macroscopic fluorescence imaging, which is commonly employed for studying brain signaling by means of genetically encoded calcium indicators.^[^ ^31^ ^]^

Accurate registration between OAT and MRI is a nontrivial task owing to the distinctly different contrast mechanisms provided by the two modalities and the lack of common landmarks. In this work, we developed a semi‐automatic image coregistration protocol between OAT and different MRI sequences using SPM12 software. Although accurate coregistration was achieved, substantial efforts were spent on alignments in the pre‐registration phase. This is partially ascribed to the relatively low quality of images acquired by the label‐free MRA method using the FLASH sequence. Alternatively, contrast‐enhanced MRA can be exploited by administering gadolinium‐based contrast agents to improve vascular contrast and thus facilitate image registration with OAT.^[^ ^32^ ^]^ Deep learning algorithms have recently been reported for multimodal data coregistration.^[^ ^26^ ^]^ However, extensive manual annotations are generally required to efficiently train those models, which are not currently available for multi‐modal OAT and MRI datasets.

In summary, our work is the first to report on concurrent in vivo observations of brain activity with functional OAT and MRI. Multiple hemodynamic parameters were detected simultaneously, facilitating cross‐validation and comprehensive readings of complex brain activity patterns. The hybrid imaging platform may find broad applicability for studying neurovascular and neurometabolic coupling mechanisms and related diseases.

## 4 Experimental Section

### Hybrid Magnetic Resonance Optoacoustic Tomography System

The hybrid MROT imaging system (Figure 1b) was based on a customized MRI‐compatible spherical matrix transducer array (Imasonic SAS, Voray, France), MRI‐compatible fiber bundle (CeramOptec GmbH, Bonn, Germany), and a customized RF coil integrated into a 3D‐printed animal holder and inserted into a high‐field MRI scanner (BioSpec 94/20, Bruker BioSpin, Germany). Optoacoustic signals were excited with a short‐pulsed (<10 ns) laser beam generated by an optical parametric oscillator (OPO) laser (Spit‐Light, Innolas Laser GmbH, Germany). The laser wavelength was rapidly swept between five distinct wavelengths (700, 730, 755, 800, and 850 nm) on a per‐pulse basis at 50 Hz pulse repetition frequency (PRF). The pulsed light was delivered by means of a fiber bundle with the per‐pulse energy at the output measured to be 8 mJ. The generated optoacoustic signals were detected by a spherical transducer array consisting of 384 individual piezocomposite elements having 5 MHz central frequency and >80% detection bandwidth (at −6 dB). The elements were distributed on a 40 mm radius spherical surface covering an angular aperture of 130° (solid angle of 1.15*π* steradians). The imaged animal was placed in its prone position on a 3D‐printed cylindrically shaped holder with the nose pointing downwards and the brain located in the center of the spherical array geometry. Two loops of saddle RF coils were integrated on either side of the mouse head holder with their polarization perpendicular to the main magnetic field (Figure 1b). To facilitate acoustic coupling, the volume between the active surface of the transducer array and the mouse head was filled with heavy water (deuterium oxide) enclosed inside a customized polyetheretherketone (PEEK) cap attached to the transducer array. The cap had a central 36 mm diameter aperture covered with a thin layer of an optically and acoustically transparent polyethylene membrane, which was in contact with the mouse head. The detected optoacoustic signals were simultaneously sampled with a custom‐made data acquisition system (DAQ, Falkenstein Mikrosysteme GmbH, Germany) at 40 Megasamples per second (Msps) and transmitted to a PC via Ethernet. The MROT data acquisition was triggered by the laser output trigger signal and synchronized with the stimulation paradigm using an external trigger device (Pulse Pal V2, Sanworks, USA).

### MRI Data Acquisition

MRI acquisitions were conducted on a 9.4T Bruker Biospec 94/20 small animal MR system (Bruker BioSpin MRI, Ettlingen, Germany) using custom‐made RF coils. ParaVision 6.0.1 was used as the user interface. MRA images were acquired to facilitate image coregistration with OAT using a FLASH sequence: FOV  =  20 × 20 mm^2^, matrix dimension (MD)  =  256 × 256, 20 slices from the brain surface to deeper regions, slice thickness = 0.3 mm, repetition time (TR)  =  13 ms, echo time (TE)  =  1.8904 ms, number of averages (NA)  =  4. Subsequently, a T1‐weighted scan in the coronal plane was acquired as anatomical reference for the fMRI data using a FLASH sequence: FOV  =  20 × 10 mm^2^, MD  =  160 × 80, 11 slices from anterior to posterior, slice thickness = 0.7 mm, TR  =  500 ms, TE  =  2.1366 ms, NA =  8. Prior to fMRI data acquisition, the local field homogeneity was optimized using the acquired B0 field maps. BOLD data were acquired using gradient‐echo echo‐planar imaging (GE‐EPI) sequence: FOV  =  20 × 10 mm^2^, MD  =  80 × 40, yielding an in‐plane voxel dimension of 250 × 250 µm^2^, 11 slices from anterior to posterior, slice thickness = 0.7 mm, flip angle (FA)  =  60°, TR  =  995 ms, TE  =  12 ms, NA  =  1, yielding an effective temporal resolution of 1 s for the volumetric acquisitions.

### OAT Data Acquisition

Volumetric time‐lapse OAT data were acquired at 700, 730, 755, 800, and 850 nm excitation wavelengths. For each wavelength, corrupted frames due to RF‐induced interference were first identified in the acquired sinograms by subtracting the nominal mean values of each channel. Specifically, if the L1 norm of the residuals was beyond a given threshold, the sinogram was regarded as corrupted. Sinogram restoration was then performed by downsampling the volume rate to 1 Hz to match the fMRI volume rate. In this step, pixel‐wise processing was performed to form the new sinograms, and outliers were rejected before averaging the consecutive frames. After sinogram restoration, the raw signals were bandpass filtered between 0.1 and 8 MHz, and image reconstruction was performed with a graphics processing unit (GPU)‐based filtered back‐projection algorithm.^[^ ^33^ ^]^ To obtain accurate reconstructions, different speeds of sound values were considered for the heavy water medium (≈1400 m s^−1^) and the mouse tissue (≈1530 m s^−1^). A voxel size of 100 × 100 × 100 µm^3^ and FOV of 8 × 8 × 4 mm^3^ were used. The images were normalized with the laser pulse energy readings at the corresponding wavelengths. To compensate for the signal intensity decay with depth, the images were further normalized with an exponential light attenuation function. Finally, linear spectral unmixing was performed to retrieve the distribution of HbO and HbR in the brain. HbT was calculated as the sum of HbO and HbR while sO_2_ was estimated as the HbO to HbT ratio.^[^ ^16^ ^]^

### Image Coregistration between MRI and OAT

OAT images mainly depicted major brain vessels owing to the strong optical absorption by whole blood (Figure 1d). The vascular OAT contrast was thus exploited for coregistration with MRA (Figure 1e). Given the concurrent 3D measurement from both modalities, the OAT image was aligned to the MRA image using a rigid transformation. The alignment was further optimized using SPM12 based on mutual information (Figure 1f). The same procedure was adopted for aligning MRA to the T1‐weighted scan, which finally resulted in coregistration between the OAT and fMRI images.

A detailed coregistration protocol can be found in the Supplementary protocol for OAT‐MRI image coregistration.

### Spatial Resolution Characterization

The OAT spatial resolution was characterized by imaging a cluster of ≈40 µm diameter absorbing microspheres (Cospheric BKPMS‐1.2 38–45 um) at 800 nm excitation wavelength. Two hundred consecutive volumetric image frames were reconstructed and averaged to achieve a clear image with a high SNR. The spatial resolution was estimated from the 1D image profiles, resulting in 163.5 and 163.2 µm along the lateral (*x*,*y*) and axial (*z*) dimensions, respectively (Figure S10, Supporting Information). For MRI, the spatial resolution performance was principally determined by the voxel size of the volumetric image, which greatly varied for the different sequences depending on the selected matrix size, the FOV, and the slice thickness, as described in the MRI data acquisition section.

### Animal Models

Athymic female nude mice (Foxn1^nu^, Charles River Laboratories, USA, 6‐week‐old, *n* = 6) were imaged in this study. The animals were housed in individually ventilated, temperature‐controlled cages under a 12‐h reversed dark/light cycle. Pelleted food (3437PXL15, CARGILL) and water were provided ad‐libitum. Mouse housing, handling, and experimentation were performed in accordance with the Swiss Federal Act on Animal Protection and were approved by the Cantonal Veterinary Office Zurich (license #ZH161/18).

### In Vivo Imaging

All mice were anesthetized for the in vivo imaging experiments. Anesthesia was inducted with intraperitoneal (IP) injection of a mixture of ketamine (100 mg kg^−1^ body weight, Pfizer) and xylazine (10 mg kg^−1^ body weight, Bayer). The injected bolus was administered in two steps with a 5 min gap to prevent cardiac depression. Maintenance injection was administered i.p. every 45 min. It consisted of a bolus of a mixture of ketamine (25 mg kg^−1^ body weight) and xylazine (1.25 mg kg^−1^ body weight) administered in a single step. Both the scalp and skull of the mice were kept intact for the experiments, while the fur on the mouse head was removed with shaving cream. Imaging was performed by placing each mouse onto the 3D‐printed mouse bed in a prone position with the nose pointing downwards (Figure 1b). Ultrasound gel mixed with heavy water was applied on the mouse head to ensure optimal acoustic coupling through the transparent polyethylene membrane while minimizing the signal distortion in MRI data acquired with different sequences. The mouse head was immobilized using a custom 3D‐printed stereotactic frame. After positioning the mouse, the platform containing both the ultrasound array and the RF coil was inserted into the bore of the MRI scanner. During the experiment, an oxygen/air mixture (0.2/0.8 L min^−1^) was provided through a breathing mask. Body temperature and respiration were continuously monitored during data acquisition with an MRI‐compatible rectal thermometer and a pneumatic pillow (SA Instruments, USA). The heart rate and SpO_2_ were monitored in real‐time with an MRI‐compatible mouse paw pulse oximeter working with a PhysioSuite (Kent Scientific Corporation, USA). The body temperature was kept around 37 °C with a temperature‐controlled water heating unit.

### Sensory Stimulation

Sensory stimulation was performed following an established BOLD fMRI stimulation paradigm.^[^ ^26^ ^]^ Briefly, unipolar rectangular electric pulses of 0.5 ms duration and 0.5 mA intensity were applied to the left forepaw at 4 Hz stimulus frequency, 20 s onset time, and 80 s burst intervals, that is, 100 s stimulus repetition cycle (Figure 1c). The electric signals were generated using a stimulus isolator device (Model A365R, World Precision Instruments, USA) fed by an external trigger (Pulse Pal V2, Sanworks, USA). Each stimulation sequence included nine stimulation cycles and lasted 980 s during which the first 80 s were reserved for baseline recording. The stimuli were synchronized with the excitation light pulses and the concurrent MROT data acquisition. After the experiments, the animals were euthanized while still under anesthesia.

### Statistical Analysis

Functional MRI and OAT data analysis were performed using Matlab (version R2019b, Mathworks, Natick, MA, USA) and the open‐source SPM software (version 12, Functional Imaging Laboratory, Welcome Trust Centre for Human Neuroimaging, University College London).

The following pre‐processing steps were performed to improve sensitivity for detecting brain activation. Note that the first 5 s of the fMRI and OAT scans were discarded prior to pre‐processing to remove the dummy scans from fMRI and corresponding OAT frames acquired during the laser warming up state. In the first step, the reconstructed images were aligned to the mean image of the sequence using the Realign (estimate) function of SPM12 for motion correction. Specifically, six motion parameters (i.e., three translations and three rotations) were obtained to perform a rigid‐body transformation. In the second step, T1‐weighted EPI and OAT images were coregistered as described above. All scans were resliced to a voxel size of 0.2 × 0.2 × 0.2 mm^3^. The Allen Mouse Brain Atlas (Allen Institute for Brain Science, http://mouse.brain‐map.org/) was spatially normalized to the individual T1‐weighted images using both affine and nonlinear transformations. Finally, the functional image data were smoothed by spatial convolution with a 0.6 mm FWHM Gaussian kernel. The complete pre‐processing data analysis pipeline is illustrated in Figure S11, Supporting Information.

The *t*‐value maps from different channels (i.e., HbO, HbR, HbT, sO_2_, and BOLD) were obtained for each animal with GLM analysis. The first‐order canonical basis set with the convolution of the hemodynamic response function (HRF) and the stimulation paradigm was used as a regressor. Default HRF parameters in SPM12 that are optimized for human studies were modified with parameters optimized with a characteristic small animal BOLD response.^[^ ^34^ ^]^ Note that six motion parameters obtained in the motion correction step were also regressed to further reduce motion artifacts.^[^ ^35^, ^36^ ^]^ A high‐pass filter with a cut‐on frequency of 1/200 Hz was used to remove slow signal drifts. The activation map in each channel was subsequently obtained by applying an initial threshold of uncorrected *p* < 0.001 to the *t*‐value map and voxels were considered statistically significant after FWE correction at *p* < 0.05 with one‐sided *t*‐tests.

For visualization, the activation map for each animal was overlaid on the corresponding T1‐weighted image and Allen mouse brain atlas (Figure 2a and Figure S5, Supporting Information). Activation maps from HbO, HbR, and BOLD were superimposed and overlaid on the brain atlas for spatial correlation analysis of the stimulus‐evoked response (Figure 2b). The time courses were analyzed within a time window covering 10 s pre‐stimulation, 20 s stimulation onset, and 60 s post‐stimulation. The baseline signal of each stimulation cycle was calculated by averaging the signals in the 10 s pre‐stimulation time window. The fractional signal changes in each stimulation cycle were calculated as (signal(*t*)–baseline)/baseline, where *t* is time. The activation time course was obtained by averaging all the stimulation cycles (Figure 2c). Statistics on activation intensity and TTP were further performed based on the activation time courses from each mouse (Figure 3b,c). Maximum *t*‐values (*t*_max_) were extracted from the contralateral primary somatosensory cortex (S1). Statistical parameters of the *t*_max_ values were displayed (Figure 3d). To further investigate the relationship between the functional activation maps of different hemodynamic components, the *t*_max_ within the S1 brain region was plotted separately for each component (Figure 3e–h). Pearson correlation coefficients (*r*) were calculated for *t*_max_ values between the BOLD activation maps and other components recorded by OAT.

## Conflict of Interest

The authors declare no conflict of interest.

## Authors Contribution

D.R. conceived the concept. Z.C. devised the hybrid system with the help of M.‐A.A. and X.L.D‐B.. Z.C., I.G., and M.‐A.A. performed the animal experiments with the help of Y.‐H.L., X.L.D.‐B., and R.N. Z.C. and I.G. performed image reconstruction and data analysis. X.L.D.‐B., R.N., and Y.‐H.L. provided guidance on experimental procedures and data analysis. D.R. supervised the work. All authors contributed to writing and revising the manuscript.

## Supporting information

Supporting information

## Data Availability

The data that support the findings of this study are available from the corresponding author upon reasonable request.
