# Supplementary material for: Simultaneous Functional Magnetic Resonance and Optoacoustic Imaging of Brain‐Wide Sensory Responses in Mice
Source: Adv Sci (Weinh). 2022 Nov 27;10(3):2205191. doi: 10.1002/advs.202205191 (PMC9875624; doi:10.1002/advs.202205191)
Supplement: Supplementary file 1 — Supporting information [file ADVS-10-2205191-s001.pdf]

ADVANCED SCIENCE
Open Access

Supporting Information

for *Adv. Sci.*, DOI 10.1002/advs.202205191

Simultaneous Functional Magnetic Resonance and Optoacoustic Imaging of Brain-Wide Sensory Responses in Mice

*Zhenyue Chen, Irmak Gezginer, Mark-Aurel Augath, Yu-Hang Liu, Ruiqing Ni, Xosé Luís Deán-Ben and Daniel Razansky**

Supplementary Information for

Simultaneous functional magnetic resonance and optoacoustic imaging of brain-wide sensory responses in mice

Zhenyue Chen[1,2], Irmak Gezginer[1,2], Mark-Aurel Augath[1,2], Yu-Hang Liu[1,2], Ruiqing Ni[1,2,3], Xosé Luís Deán-Ben[1,2], and Daniel Razansky[1,2,3,*]

[1]Institute for Biomedical Engineering and Institute of Pharmacology and Toxicology, Faculty of Medicine, University of Zurich, Switzerland

[2]Institute for Biomedical Engineering, Department of Information Technology and Electrical Engineering, ETH Zurich, Switzerland

[3]Zurich Neuroscience Center (ZNZ), Switzerland

*Correspondence
Daniel Razansky, Institute for Biomedical Engineering, Wolfgang-Pauli-Str. 27, 8093 Zurich, Switzerland
Email: daniel.razansky@uzh.ch

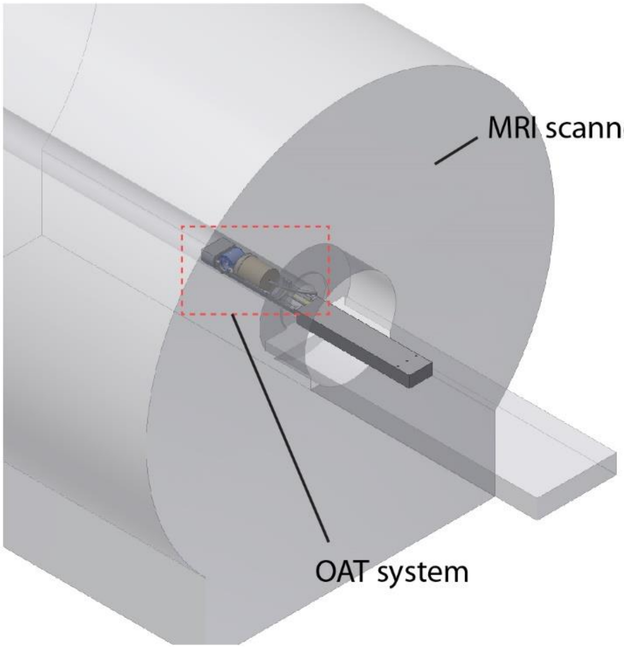

Supplementary Figure 1 | Schematic drawing of the hybrid magnetic resonance optoacoustic tomography (MROT) system.

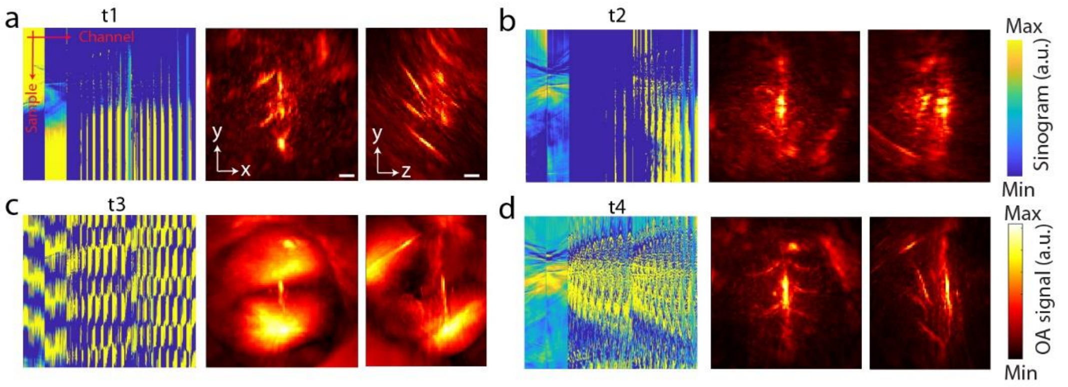

Supplementary Figure 2 | Representative raw OAT sinograms and corresponding OAT image reconstructions shown in x-y and y-z views. **a-c** display three greatly corrupted frames while **d** displays an uncorrupted frame. t1 - t4: representative time instances. Scale bar: 1mm.

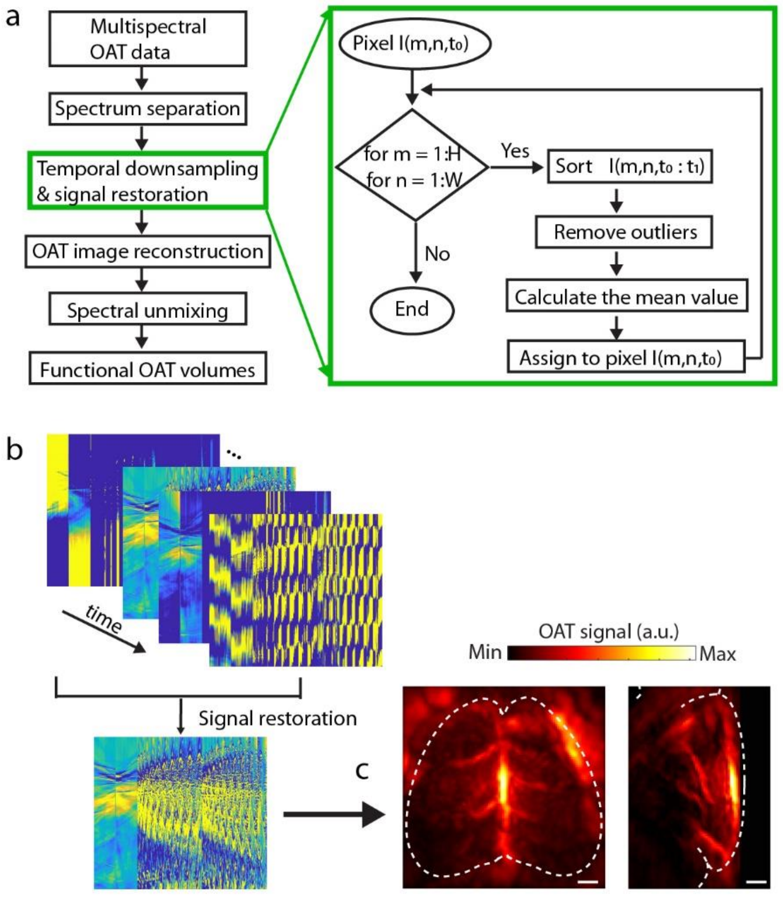

Supplementary Figure 3 | OAT sinogram restoration and image reconstruction. **a** OAT image reconstruction pipeline. The sinogram restoration proceedure is shown in the green box. **b** Illustration of sinogram restoration and **c** corresponding OAT reconstruction shown in x-y and y-z views. Scale bar: 1mm.

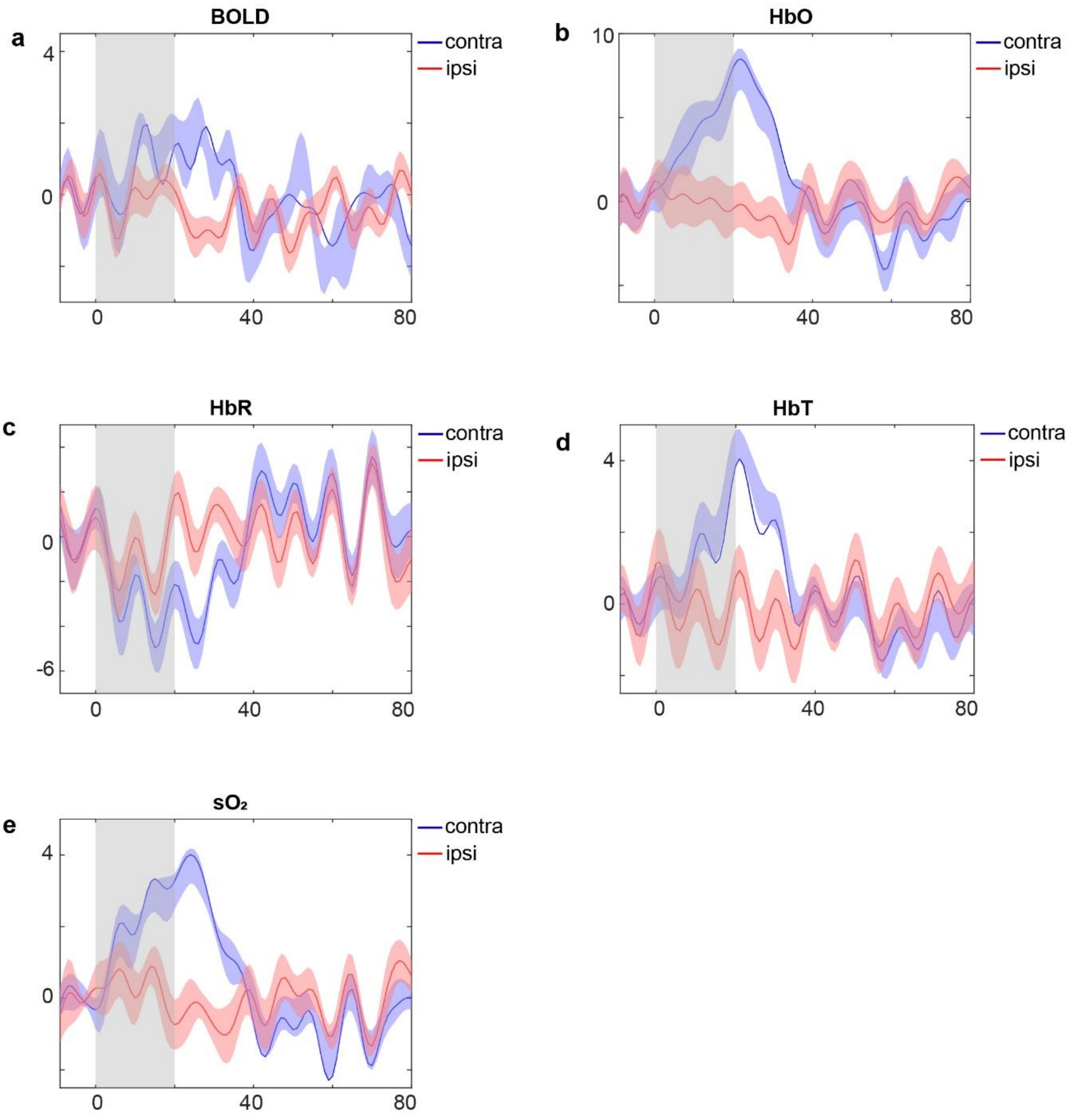

Supplementary Figure 4 | Averaged relative signal intensity changes calculated for the different hemodynamic components recorded by fMRI and OAT during sensory stimulation in contralateral and ipsilateral S1FL brain region. **a-e** Activation time curves (± s.e.m) in the contralateral and ipsilateral S1FL regions. Activation time curve for each component was computed for 0.4 x 0.4 x 0.4 mm^3 ROIs located in the contralateral and ipsilateral S1FL regions. Gray bars indicate stimulation period.

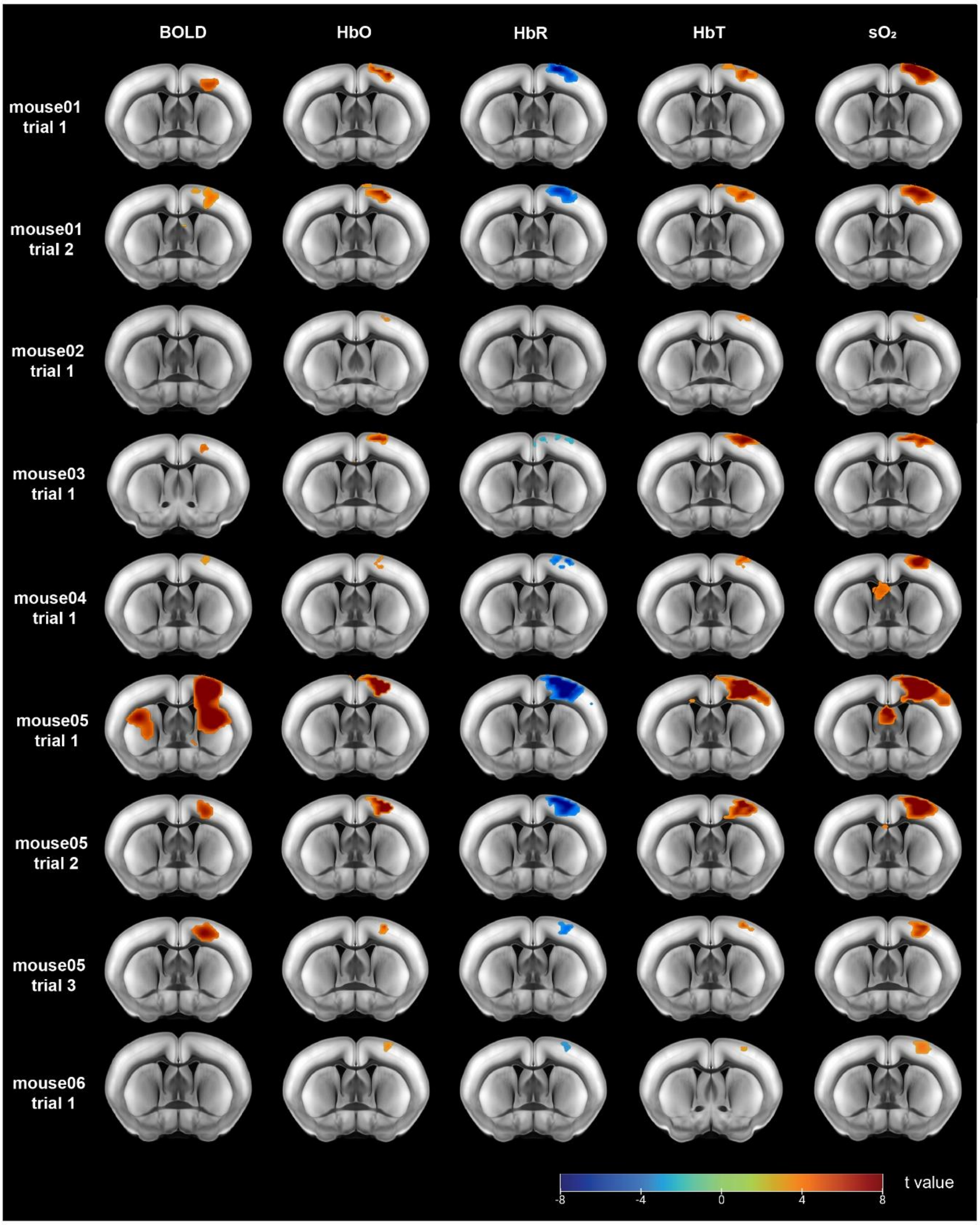

Supplementary Figure 5 | Statistically significant activations detected across different hemodynamic components in response to the electrical forepaw stimulation paradigm in all the mice. Activation maps are shown for a single coronal slice and overlaid with Allen Mouse Brain Atlas. Significant responses were observed in contralateral S1FL and M1 brain regions from the BOLD, HbO, HbR, HbT and sO2 responses. No statistically significant response was detected from the BOLD response for mouse02, trial1 and mouse06, trial1, and from the HbR response for mouse02, trial1. The left side of the image corresponds to the left hemisphere. $p < 0.05$, family-wise error corrected.

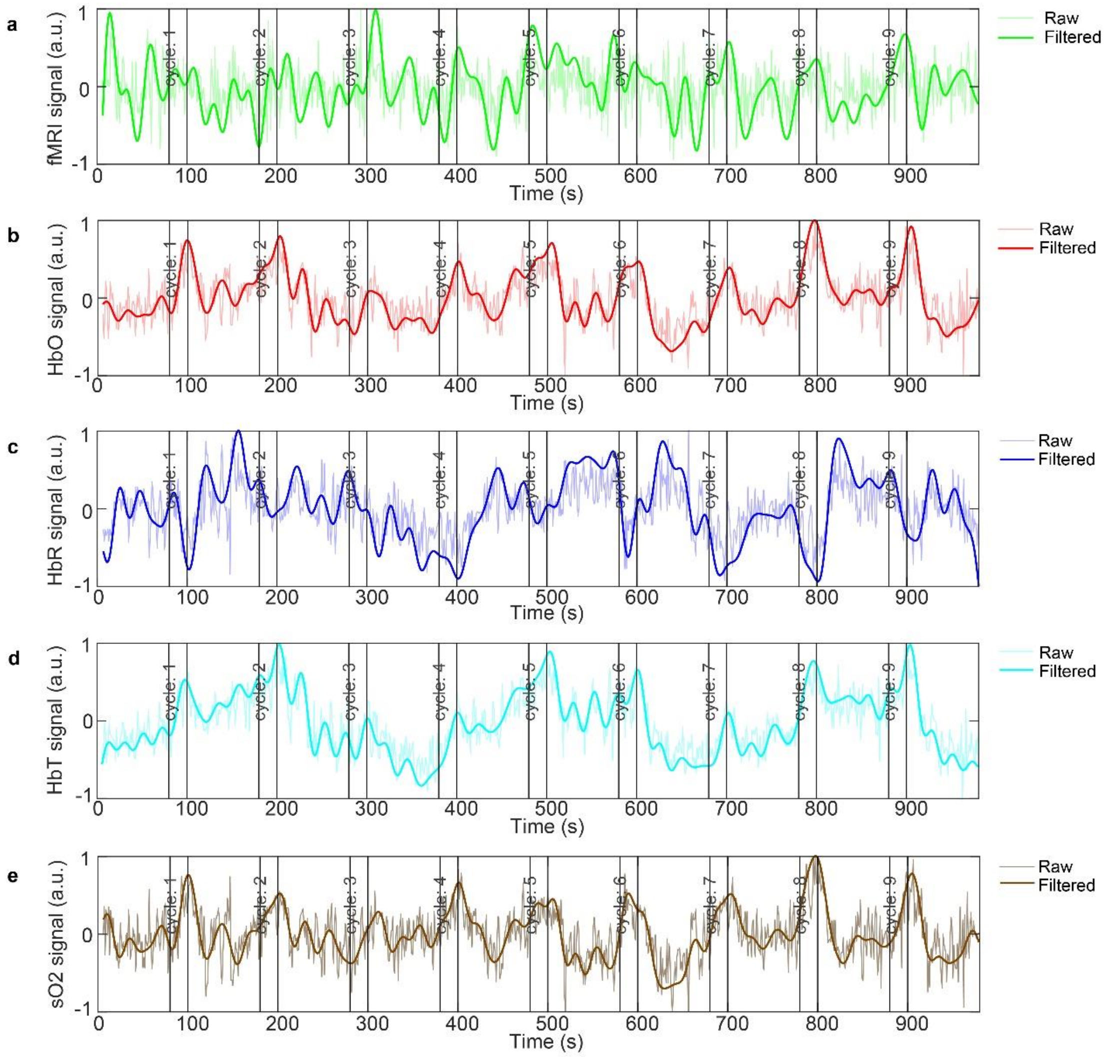

Supplementary Figure 6 | Full-length time-courses for the different hemodynamic components extracted from the ROI located in contralateral S1FL region for an exemplary stimulation trial shown in Fig. 2. Both raw and low-pass filtered signals are shown.

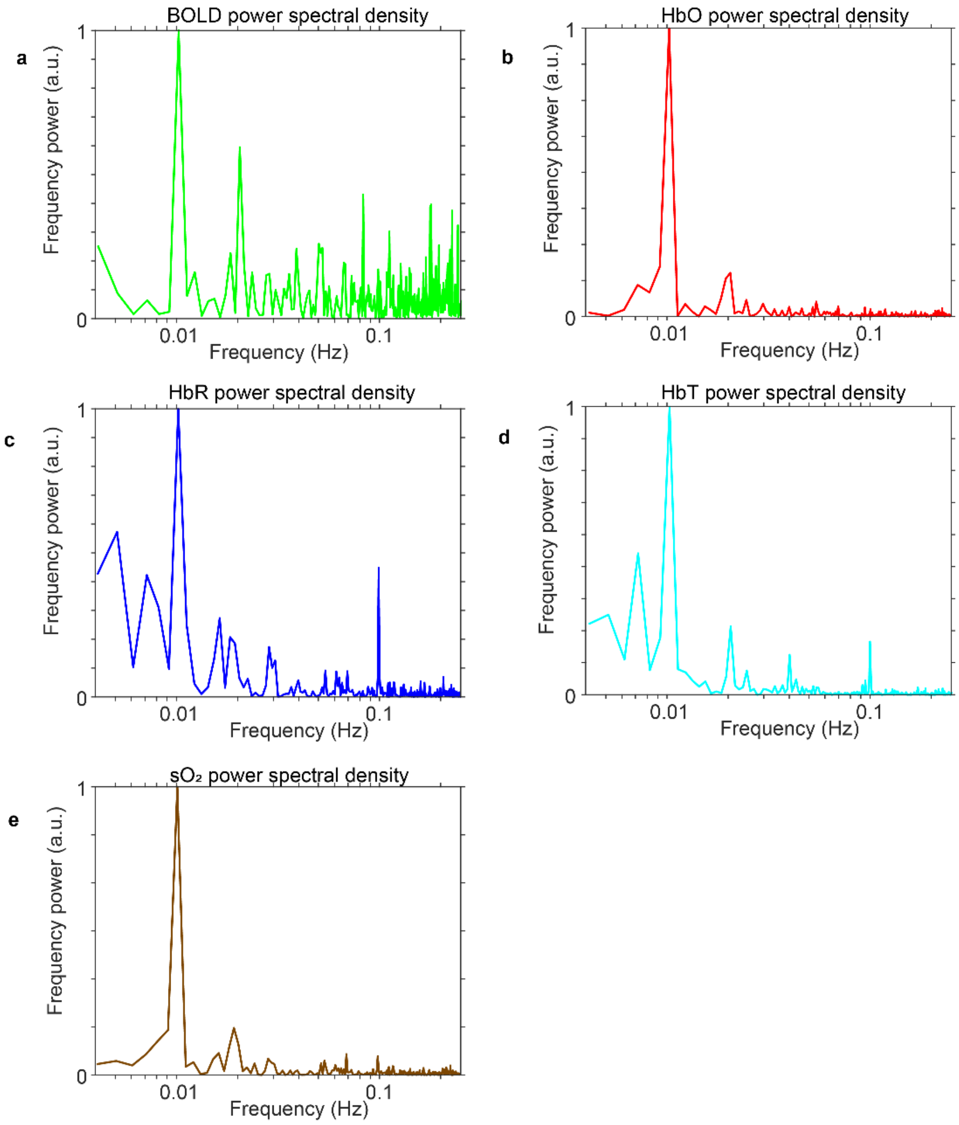

Supplementary Figure 7 | Power spectral density of the full-length time-courses shown in SFig. 6. Note that the peak frequency of 0.01 Hz detected in all the hemodynamic components coincides with the boxcar frequency of the stimulation paradigm.

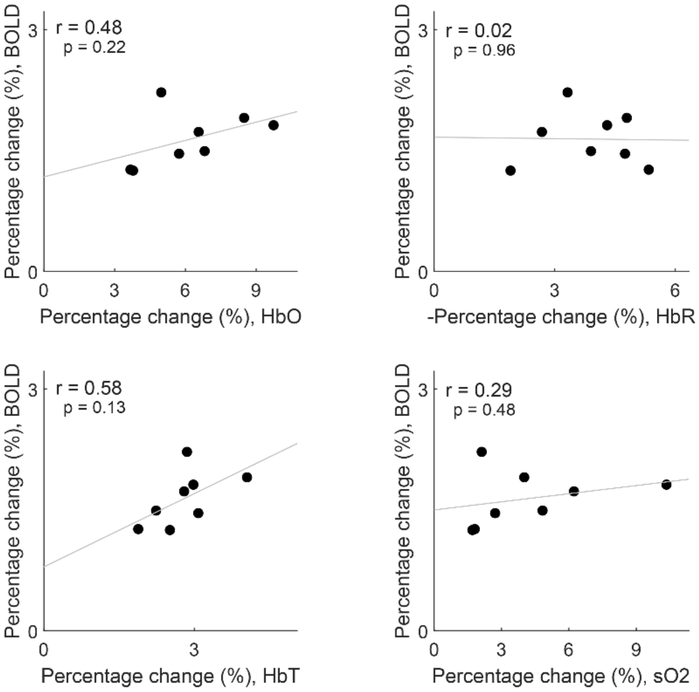

Supplementary Figure 8 | Scatter plots showing the relationship between activation intensity values computed from BOLD and the different hemodynamic components recorded with OAT. Each of the stimulation trials is represented in the scatter plot with a dot, except one outlier that was removed. Pearson correlation coefficients (r) were calculated for the plots. Gray lines represent linear fits of the data.

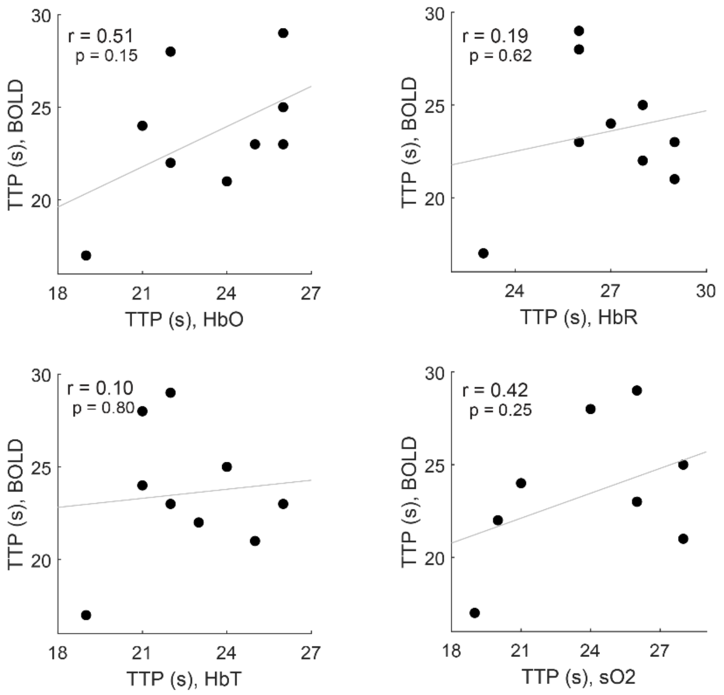

Supplementary Figure 9 | Scatter plots showing the relationship between TTP values computed from BOLD and different OAT-extracted components. Each of the stimulation trials is represented in the scatter plot with a dot. Pearson correlation coefficients (r) were calculated for the plots. Gray lines represent linear fits of the data.

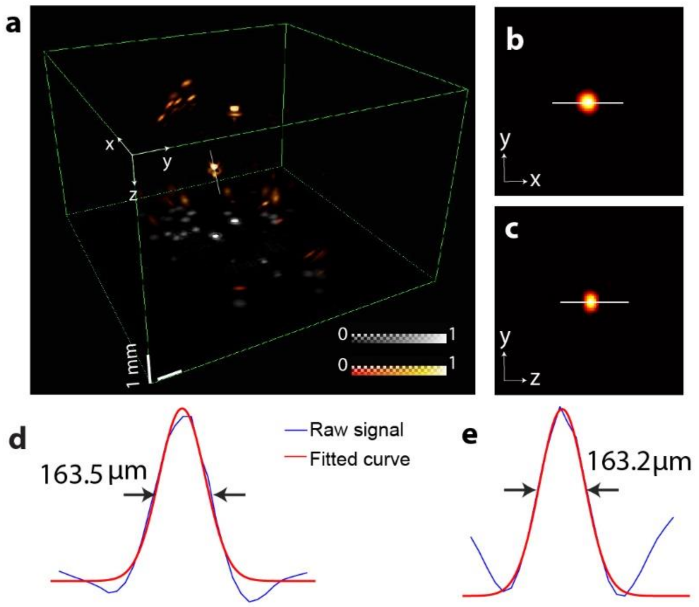

Supplementary Figure 10 | Spatial resolution characterization of OAT by imaging a cluster of microspheres with diameters ranging between 38-42 μm. **a** Volumetric OAT image reconstructed from the microsphere phantom. **b**,**c** Slice image of the selected microsphere in x-y and y-z views, respectively. **d**,**e** One-dimensional signal profiles along the white lines indicated in **b** and **c**, depicting the lateral and axial resolutions of ~163.5 μm and ~163.2 μm, respectively.

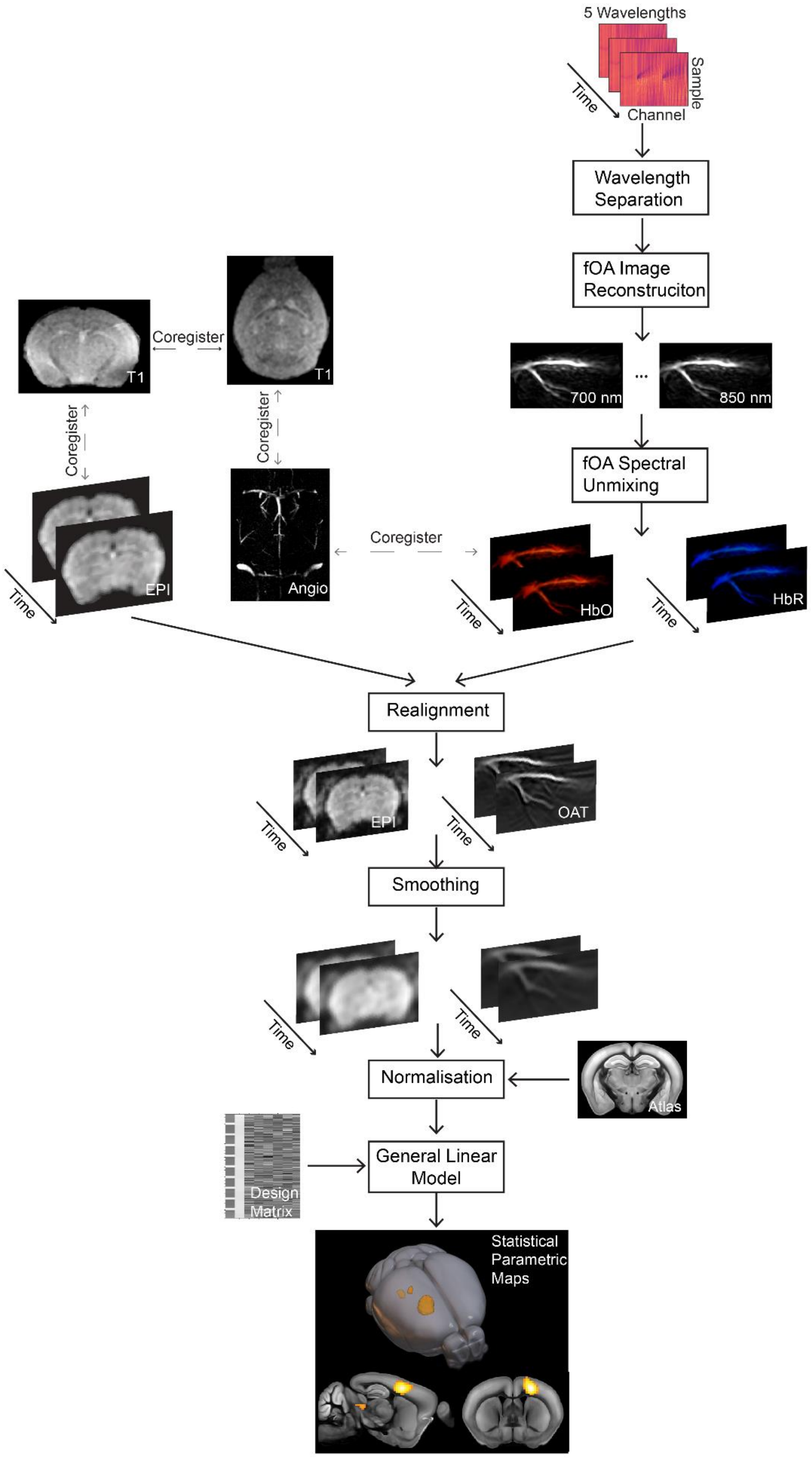

Supplementary Figure 11 | Illustration of the data analysis pipeline for the multi-modal functional imaging data .
